# Quality appraisal of clinical practice guidelines for fertility preservation in patients with breast cancer: a systematic review using AGREE II and AGREE-REX

**DOI:** 10.3389/fmed.2026.1930322

**Published:** 2026-10-07

**Authors:** Wenjuan Yan, Lili Wang, Hongjuan Zhou, Lili Zhao, Yunfei Zhang

**Affiliations:** 1 Department of Nursing, Women and Children's Hospital, School of Medicine, Xiamen University, Xiamen, Fujian, China; 2 Digestive Endoscopy Center, Quanzhou First Hospital Affiliated to Fujian Medical University, Quanzhou, Fujian, China; 3 School of Nursing, Li Ka Shing Faculty of Medicine, University of Hong Kong, Hong Kong SAR, China; 4 Department of Interventional Radiology Center, School of Medicine, Xiamen Cardiovascular Hospital of Xiamen University, Fujian Branch of National Clinical Research Center for Cardiovascular Diseases, Xiamen, Fujian, China

**Keywords:** AGREE II, AGREE-REX, breast cancer, fertility management, guidelines, systematic review

## Abstract

**Objective:**

This review utilizes the AGREE II instrument to evaluate the methodological rigor and the AGREE-REX instrument to evaluate the implementability of recommendations within clinical practice guidelines for fertility preservation in patients with breast cancer. This is the first study to comprehensively appraise related guidelines using both officially recognized instruments. The findings aim to inform clinical application by identifying high-quality and implementable guidelines.

**Methods:**

By June 2026, a systematic search was conducted across seven electronic databases, six guideline repositories, and relevant breast cancer society websites. Guidelines were screened according to predefined criteria, and independent quality assessments were performed using AGREE II and AGREE-REX. Inter-rater reliability was evaluated via the intraclass correlation coefficient (ICC).

**Results:**

Twelve guidelines were included. The AGREE II domain scores (mean standardized percentages) showed significant variations: Scope and Purpose (72.98%), Editorial Independence (71.81%), Clarity of Presentation (70.05%), Applicability (69.33%), Rigor of Development (55.30%), and Stakeholder Involvement (54.78%), highlighting critical gaps in patient participation and evidence updating mechanisms. Based on AGREE II and AGREE-REX evaluations, five guidelines were classified as Grade A (strongly recommended), four as Grade B, and three as Grade C.

**Conclusion:**

The five Grade A guidelines predominantly originated from Western countries. Prior to clinical implementation, they require cultural adaptation, language translation, and local validation to align with regional healthcare systems and patient values. Future guideline development should prioritize stakeholder integration, dynamic updating mechanisms, and resource allocation assessments to enhance clinical applicability.

**Systematic Review Registration:**

https://www.crd.york.ac.uk/PROSPERO/, PROSPERO CRD42023464394.

## 1 Introduction

Breast cancer is defined as a malignant tumor originated from abnormal proliferation of mammary epithelial cells (1). Early clinical manifestations are characterized by breast lumps and nipple discharge. When left untreated, tumor progression can result in multi-organ dysfunction and even life-threatening outcomes. Epidemiological evidence (2). has demonstrated a continuous increase in the incidence of breast cancer among reproductive-aged women globally, with nearly 60% of countries reporting it as the leading cause of cancer-related mortality in females (3). Despite significant improvements in survival rates achieved by comprehensive treatments including surgery, chemotherapy, and radiotherapy (4), treatment-related adverse effects such as ovarian dysfunction and fertility impairment remain largely unavoidable.

The concept of “oncofertility” which was proposed in 2007 (5), emphasizes the necessity of multidisciplinary collaboration for the development of personalized fertility preservation strategies. With the advancement of society and the trend towards delayed childbearing, a significant number of patients are diagnosed with breast cancer prior to the completion of their family planning. Nevertheless, concerns regarding disease prognosis and the health of offspring frequently compel these patients to relinquish their reproductive aspirations. Moreover, anxiety associated with fertility may have a detrimental impact on treatment adherence. According to studies (6), patients whose fertility needs remain unfulfilled are at an elevated risk of experiencing psychological distress, including depression. Consequently, standardized fertility preservation counseling is deemed crucial for patients with breast cancer.

Currently, notable discrepancies are observed in fertility preservation recommendations across international clinical practice guidelines (CPGs). Regarding the timing of counseling, fertility counseling during anticancer therapy is advised by the European Society for Medical Oncology (ESMO) (7), while initiation immediately after diagnosis is recommended by the Korean Society for Fertility Preservation (KSFP) (8). As for the post-treatment pregnancy interval, a minimum 6-month wait after therapy is suggested by the National Comprehensive Cancer Network (NCCN) (9), whereas this interval is extended to 12 months by the European Society of Human Reproduction and Embryology (ESHRE) (10). Such inconsistencies may lead to challenges in clinical decision-making and further intensify patients’ psychological distress. To address these issues and gaps, we assessed the methodological quality of relevant guidelines to provide evidence-based support for clinical practice.

## 2 Methods

### 2.1 Design and registration

This systematic review utilizes the Appraisal of Guidelines for Research & Evaluation II (AGREE II) instrument to assess the methodological rigor and the Appraisal of Guidelines for Research & Evaluation-Recommendations Excellence (AGREE-REX) instrument to evaluate the quality and implementability of recommendations within clinical practice guidelines for fertility preservation in patients with breast cancer. The study protocol is registered on PROSPERO (CRD42023464394). The completed PRISMA checklist is provided as Supplementary Material 1.

### 2.2 Search strategy and selection of guidelines

A comprehensive literature search was performed using a combination of controlled vocabulary (MeSH/Emtree terms) and free-text keywords, including “Breast cancer,” “Clinical practice guidelines,” and “Fertility preservation.” The search was designed to cover records from the inception of each database through June 1, 2026, across multiple information sources. The search strategy encompassed three categories of resources: (a) General databases, including PubMed, Cochrane Library, Embase, Web of Science, China National Knowledge Infrastructure (CNKI), Condition-Based Maintenance (CBM), and Wanfang Data; (b) Specialized guideline repositories, such as NCCN, National Guideline Clearinghouse (NGC), Guidelines International Network (GIN), New Zealand Guidelines Group (NZGG), Chinese Guideline Clearinghouse, and Medlive; (c) Official websites of relevant professional societies, including the European Society of Breast Cancer Specialists (EUSOMA), European Society for Medical Oncology (ESMO), ESHRE, KSFP, American College of Obstetricians and Gynecologists (ACOG), Society of Obstetricians and Gynaecologists of Canada (SOGC), UK National Institute for Health and Care Excellence (NICE), Royal College of Obstetricians and Gynaecologists (RCOG), and the Breast Surgery Group of Chinese Medical Association. The complete search strategies for all databases, with PubMed as an example, are presented in Supplementary Material 2.

The inclusion criteria were as follows: (a) CPGs published in Chinese or English; (b) CPGs explicitly targeting breast cancer patients and containing fertility preservation recommendations; (c) given the scarcity of guidelines exclusively addressing fertility preservation in breast cancer, we also included broader guidelines covering fertility preservation across multiple cancer types, provided that they contained a dedicated section or specific recommendations applicable to breast cancer patients; (d) when updated versions expanded upon earlier editions, both versions were included to facilitate comparative analysis.

The exclusion criteria were as follows: (a) non-guideline documents, like expert consensus and position statements; (b) guideline interpretations or commentary; (c) translated versions of guidelines that had already been included.

The literature screening process adhered to rigorous systematic review methodology. Duplicates were first removed, and clearly irrelevant records were subsequently excluded based on title and abstract by two independent reviewers (Yan WJ and Wang LL) using Covidence software. For potentially eligible guidelines, full texts were retrieved and evaluated against predefined inclusion and exclusion criteria. During this process, any discrepancies between reviewers were resolved through discussion with a third researcher (Zhou HJ).

### 2.3 Data extraction and guideline assessment

Key guideline characteristics, such as guidelines name, breast cancer-specific, publication year, country, organization, funding support, conflict of interest declaration, number of references, and version status, were independently extracted by two reviewers (Yan WJ and Zhang YF). Disagreements were discussed, analysed and resolved through the arbitration of a third researcher (Zhou HJ). For guidelines addressing multiple cancer types, only those with specific recommendations for patients with breast cancer were included. This standardized approach ensured the methodologically sound selection of CPGs while minimizing bias in the evaluation process.

The methodological quality of included guidelines was systematically assessed using the internationally recognized AGREE II and AGREE-REX instruments. Three trained evidence-based medicine experts independently conducted the evaluations. To resolve any discrepancies during the assessment process, the following hierarchical decision-making criteria were applied: (1) level of supporting evidence, (2) guideline publication date (with preference given to more recent versions), (3) authority of the developing organization.

The methodological quality and recommendation excellence of the included guidelines were systematically evaluated using two internationally validated instruments: AGREE II and AGREE-REX tools. The AGREE II tool assesses six core domains through 23 items and two global assessment questions. These domains are: (1) scope and purpose; (2) stakeholder involvement; (3) rigor of development; (4) clarity of presentation; (5) applicability, and (6) editorial independence. Each item is rated on a seven-point Likert scale, where one represents “strongly disagree” and seven represents “strongly agree”. The domain standardized scores are calculated using the formula:

$$\frac{\mathrm{Actual}\;\mathrm{score}\;-\mathrm{Minimum}\;\mathrm{possible}\;\mathrm{score}}{\mathrm{Maximum}\;\mathrm{possible}\;\mathrm{score}\;-\;\mathrm{Minimum}\;\mathrm{possible}\;\mathrm{score}}\times 100\text{\%}$$

The AGREE II instrument does not provide official cut-off values for guideline classification, so thresholds must be predefined by researchers (11). We adopted the 60%/30% thresholds based on common methodological approaches observed in prior guideline appraisal studies, in which these thresholds have been widely applied across various clinical fields (12–14). A systematic review by Hoffmann-Eßer et al. further reported that approximately one-third of AGREE II users apply cut-offs to distinguish guideline quality, with thresholds for high quality typically ranging from 50%–70% (two-step) to 60%–83% (three-step) (15). Therefore, the grading system used in this review represents a researcher-defined classification based on the distribution of scores and prior literature, rather than an official AGREE II standard. The included guidelines were classified into three quality categories:

- Grade A (Strongly Recommended): Guidelines that achieved standardized percentage scores above 60% in all six domains.
- Grade B (Recommended with Revisions): Guidelines that scored between 30% and 60% in more than three domains.
- Grade C (Not Recommended): Guidelines that received standardized percentage scores below 30% in more than three domains.

Given the absence of established threshold criteria, we performed sensitivity analyses using more stringent thresholds to further assess the robustness of our classifications (Supplementary Material 3).

The evaluation process was complemented by the AGREE-REX tool, which focuses on three critical domains of recommendation quality: (1) clinical applicability; (2) values and preferences, and (3) implementability. This nine-item instrument, similar to the AGREE II, employs a seven-point Likert scale, with one indicating “strongly disagree” and seven indicating “strongly agree”. An identical standardization method, calculated as:

$$\frac{\mathrm{Actual}\;\mathrm{score}\;-\mathrm{Minimum}\;\mathrm{possible}\;\mathrm{score}}{\mathrm{Maximum}\;\mathrm{possible}\;\mathrm{score}\;-\;\mathrm{Minimum}\;\mathrm{possible}\;\mathrm{score}}\times 100\text{\%}$$

According to the AGREE-REX manual (16), the included guidelines were systematically classified into three distinct quality categories:

- High Quality: Recommendations with overall mean scores exceeding 70%.
- Moderate Quality: Recommendations with scores between 30% and 70%.
- Low Quality: Recommendations with scores below 30%.

### 2.4 Data analysis and synthesis

A standardized database was constructed using Microsoft Excel 2024 to systematically document the fundamental characteristics of the included guidelines. To ensure data accuracy, a dual-verification mechanism was rigorously implemented. Statistical analyses were conducted using IBM SPSS Statistics 26.0. The inter-rater reliability among evaluators was evaluated through the application of the intraclass correlation coefficient (ICC), a widely recognized statistical measure for assessing the reliability of measurement instruments, reproducibility in experimental research, and consistency among researchers (17). The ICC was calculated using a two-way random-effects model for absolute agreement, with 95% confidence intervals (CIs). The interpretation of ICC values adhered to established standards: (1) an ICC value less than 0.50 was indicative of poor agreement; (2) an ICC ranging from 0.50 to less than 0.75 suggested moderate agreement; and (3) an ICC of 0.75 or higher signified good agreement.

## 3 Result

### 3.1 Selection of eligible guidelines

The initial search identified 3,529 potentially relevant records. After a rigorous, multi-stage screening process, 12 CPGs (7–10, 18–25) ultimately met the inclusion criteria and were selected for analysis. The excluded studies and reasons are detailed in Supplementary Material 4. The complete literature screening workflow and results are presented in Figure 1.

**Figure 1:**
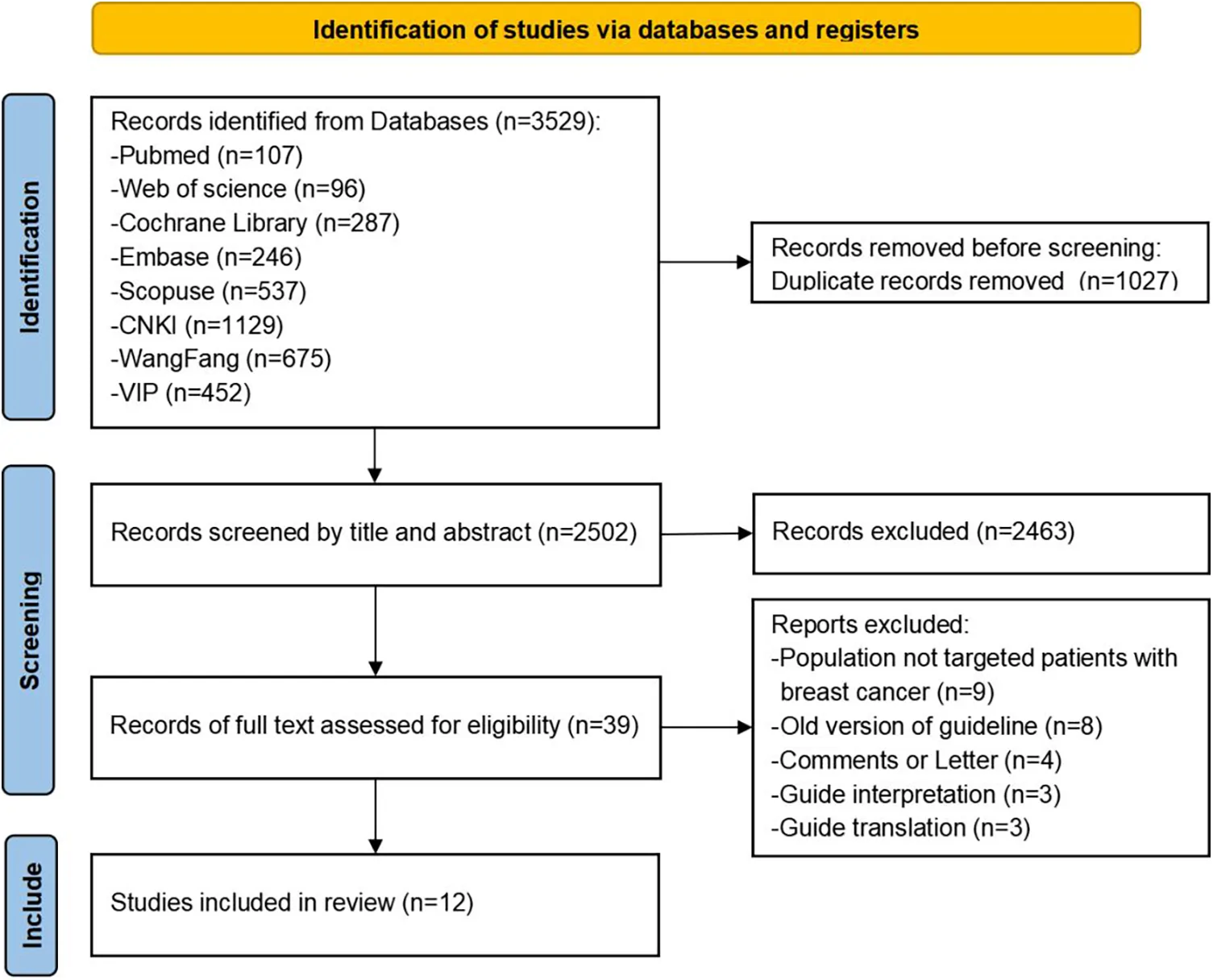
Flow chart of the literature screening process.

### 3.2 Overall characteristics of the guidelines

This review included a total of 12 guidelines (7–10, 18–25), comprising ten English-language guidelines and two Chinese-language guidelines. Geographically, the guidelines originated from: United States (*n* = 3) (9, 18, 19), United Kingdom (*n* = 3) (20–22), China (*n* = 2) (23, 24), Europe (*n* = 2) (7, 10), Canada (*n* = 1) (25), and South Korea (*n* = 1) (8). The publication years of these guidelines spanned from 2007 to 2026, with six guidelines (50%) being published or updated within the most recent five-year period. Notably, all included guidelines were relevant to patients with breast cancer, either as breast cancer-specific documents or as broader-scope guidelines containing breast cancer-specific content, ensuring direct relevance to the target population. Of the 12 included guidelines, six guidelines (50%) (8, 9, 19, 23–25) were developed specifically for breast cancer patients, while the remaining six (7, 10, 18, 20–22) addressed fertility preservation in a broader oncology or reproductive context but contained dedicated sections or specific recommendations applicable to breast cancer patients. The comprehensive characteristics of these guidelines, including development organizations, publication years, and target populations, are detailed in Table 1.

**Table 1:** Basic characteristics of inclusion guidelines.

Guideline No.& Title	BC-Specific	BC-Related Content	Year	Country	Issuing Organization	Funding	COI	Refs	Latest
Fertility preservation and post-treatment pregnancies in post-pubertal cancer patients: ESMO Clinical Practice Guidelines (7)	Yes	Broad cancer guideline; contains dedicated section on breast cancer	2020	Europe	ESMO	Yes	No	90	Yes
Fertility preservation for patients with breast cancer: The Korean Society for Fertility Preservation clinical guidelines (8)	Yes	Entire guideline focused on breast cancer	2017	Korea	KSFP	NR	No	45	Yes
NCCN Guidelines® Insights: Breast Cancer, Version 4.2026 (9)	Yes	Entire guideline focused on breast cancer; dedicated FP section	2026	USA	NCCN	Yes	No	755	Yes
ESHRE guideline: female fertility preservation (10)	Yes	Broad female FP guideline; includes dedicated breast cancer section	2020	Europe	ESHRE	Yes	No	145	Yes
Adolescent and Young Adult (AYA) Oncology, Version 2. 2026 (18)	Yes	General AYA oncology guideline; includes breast cancer-specific content	2026	USA	NCCN	Yes	No	NR	Yes
Management of Gynecologic Issues in Women With Breast Cancer (19)	Yes	Entire guideline focused on gynecologic issues in breast cancer	2012	USA	ACOG	NR	NR	166	Yes
Fertility preservation for medical reasons in girls and women: British fertility society policy and practice guideline (20)	Yes	Broad medical FP guideline; includes breast cancer-specific recommendations	2018	UK	BFS	NR	No	148	Yes
Fertility problems: assessment and treatment (21)	Yes	Broad fertility guideline; includes cancer sections with breast cancer content	2026	UK	NICE	Yes	No	NR	Yes
The effects of cancer treatment on reproductive functions (22)	Yes	Broad guideline on cancer treatment effects; includes breast cancer content	2007	UK	RCOG	NR	NR	93	Yes
Clinical practice China guidelines for breast cancer during pregnancy and lactation (2025 edition) (23)	Yes	Entire guideline focused on breast cancer during pregnancy/lactation	2025	China	CSS-CMA	NR	No	46	Yes
Clinical practice China guidelines for fertility preservation in women of childbearing age with breast cancer (2025 edition) (24)	Yes	Entire guideline focused on breast cancer FP	2025	China	CSS-CMA	Yes	No	17	Yes
No.366-Gynaecologic Management of Hereditary Breast and Ovarian Cancer (25)	Yes	Entire guideline focused on hereditary breast/ovarian cancer	2018	Canada	SOGC	Yes	No	100	Yes

BC-specific, breast cancer-specific; COI, conflict of interest; Refs, references; ESMO, European society for medical oncology; KSFP, Korean society for fertility preservation; NCCN, national comprehensive cancer network; ESHRE, European society of human reproduction and embryology; ACOG: American college of obstetricians and gynecologists; BFS: British fertility society; NICE, national institute for health and care excellence; RCOG, royal college of obstetricians and gynaecologists; CSS-CMA, Chinese society of surgery, Chinese medical association; SOGC, society of obstetricians and gynaecologists of Canada.

### 3.3 Quality appraisal of clinical practice guidelines’ methodology

The inter-rater reliability analysis yielded excellent agreement among the three evaluators, with ICC values ranging from 0.764 to 0.995 (all >0.75 and *P* < 0.001), indicating strong consistency in the assessment process. The AGREE II evaluation (26) revealed the following mean standardized domain scores (ranked from highest to lowest): Scope and Purpose (72.98%), Editorial Independence (71.81%), Clarity of Presentation (70.05%), Applicability (69.33%), Rigor of Development (55.30%) and Stakeholder Involvement (54.78%). Among the 12 evaluated guidelines, five guidelines were classified as Grade A (strongly recommended) (7, 9, 20, 21, 25); four guidelines were rated as Grade B (recommended with modifications needed) (18, 19, 23, 24); The remaining three guidelines were categorized as Grade C (not recommended) (8, 10, 22). Detailed evaluation results for each domain are presented in Table 2.

**Table 2:** AGREE II evaluation results.

Guideline Title	Percentage of Standardization (%)							＜30% number of fields (n)	≥60% Number of fields (n)	Recommended level	ICC
	Scope and Purpose	Stakeholder Involvement	Rigor of Development	Clarity of Presentation	Applicability	Editorial Independence	Average score				
Fertility preservation and post-treatment pregnancies in post-pubertal cancer patients: ESMO Clinical Practice Guidelines (7)	100.0	94.4	99.2	98.1	100.0	100.0	98.62	0	6	A	0.824
NCCN Guidelines® Insights: Breast Cancer, Version 4.2026 (9)	98.1	65.3	71.4	92.6	94.4	100.0	86.97	0	6	A	0.981
Fertility preservation for medical reasons in girls and women: British fertility society policy and practice guideline (20)	94.4	86.1	80.2	94.4	75.0	100.0	88.35	0	6	A	0.984
Fertility problems: assessment and treatment (21)	100.0	98.6	98.4	92.6	100.0	100.0	98.27	0	6	A	0.764
No.366-Gynaecologic Management of Hereditary Breast and Ovarian Cancer (25)	94.4	78.4	90.5	96.3	94.4	100.0	92.33	0	6	A	0.973
Adolescent and Young Adult (AYA) Oncology, Version 2.2026 (18)	40.7	51.4	38.9	64.8	31.9	61.6	48.22	0	2	B	0.975
Management of Gynecologic Issues in Women With Breast Cancer (19)	77.8	48.6	33.3	44.4	47.2	0.0	41.88	1	1	B	0.995
Clinical practice China guidelines for breast cancer during pregnancy and lactation (2025 edition) (25)	98.1	47.2	54.0	70.4	93.1	80.6	73.90	0	4	B	0.983
Clinical practice China guidelines for fertility preservation in women of childbearing age with breast cancer (2025 edition) (24)	29.6	37.5	40.5	44.4	50.0	63.9	44.32	1	1	B	0.982
Fertility preservation for patients with breast cancer: The Korean Society for Fertility Preservation clinical guidelines (8)	51.9	8.3	19.1	55.6	15.3	100.0	41.70	3	1	C	0.995
ESHRE guideline: female fertility preservation (10)	20.4	33.3	17.5	22.2	66.7	55.6	35.95	3	1	C	0.970
The effects of cancer treatment on reproductive functions (22)	70.4	8.3	20.6	64.8	63.9	0.0	38.00	3	3	C	0.992
Average score	72.98	54.78	55.30	70.05	69.33	71.81	65.71				0.952

Scope and Purpose achieved the highest average standardized percentage score of 72.98% among all domains. The ESMO guideline (7) and NICE guideline (21) received the highest scores (100%), as they clearly defined the health issues and provided detailed descriptions of target populations. However, two guidelines (10, 24) scored below 30%, with issues including unclear articulation of health problems and vague descriptions of target populations.

Stakeholder Involvement showed the lowest average standardized percentage score of 54.78% among all domains. The NICE guideline (21) achieved the highest score (98.6%), while two guidelines (8, 22) scored below 30%. The main deficiencies identified were failure to specify the professional backgrounds and responsibilities of guideline development group members and overly simplistic descriptions of target users.

Rigor of Development achieved an average standardized percentage score of 55.30%. While the ESMO guideline (7) received the highest score (99.2%), three guidelines (8, 10, 22) scored below 30% with no specification of external expert review processes and no documented update procedures. Two guidelines (8, 24) failed to describe evidence grading systems or development methodologies; and the ESHRE guideline (10) neither demonstrated linkage between evidence and recommendations nor provided clinical examples of patient functional outcomes.

Clarity of Presentation attained an average standardized percentage score of 70.05%. The ESMO guideline (7) achieved the highest score (98.1%), while only the ESHRE guideline (10) scored below 30%. Most guidelines (7, 9, 18, 20–23, 25) employed visual enhancement techniques (e.g., bold fonts, graphics, or special symbols) to emphasize key recommendations. The ESHRE guideline's (10) low performance primarily resulted from difficult identification of specific recommendations and ambiguous language formulation that hindered precise localization of crucial guidance.

Applicability achieved an average standardized percentage score of 69.33%. The ESMO (7) and NICE (21) guidelines received the highest scores (100%), while only one guideline (8) scored below 30% due to primary deficiencies in failure to clearly describe evidence quality standards and absence of dissemination-ready media formats to facilitate implementation.

Editorial Independence attained an average standardized percentage score of 71.81%. Six guidelines (7–9, 20, 21, 25) achieved perfect scores (100%), while two guidelines (19, 22) received 0% scores. The complete lack of scores was primarily attributed to failure to disclose conflicts of interest (19) and absence of funding source declarations (22).

Sensitivity analysis using stricter thresholds (70% for Grade A and 40% for Grade C) showed that 10 of 12 guidelines (83.3%) retained their original classifications. Two guidelines were downgraded: NCCN Guidelines (9) from A to B due to a marginal Stakeholder Involvement score (65.3%), and ACOG (19) from B to C due to deficiencies in Editorial Independence (0%) and Rigor of Development (33.3%). Notably, no C-grade guideline was upgraded and no A-grade guideline was downgraded to C and four of five Grade A guidelines remained unchanged. Detailed results are presented in Supplementary Material 3.

### 3.4 Comparison of key clinical recommendations across guidelines

While the AGREE II assessment revealed variations in methodological quality across guidelines, it does not capture whether the clinical recommendations themselves are consistent or divergent. To address this gap and enhance the clinical utility of this review, we systematically compared the major recommendations across the 12 included guidelines on five key clinical questions relevant to fertility preservation in breast cancer patients: (1) timing of fertility preservation counseling; (2) embryo and oocyte cryopreservation; (3) GnRH agonist use for ovarian protection; (4) pregnancy waiting period after chemotherapy; and (5) letrozole use during ovarian stimulation. Table 3 summarizes these comparisons.

**Table 3:** Comparison of key clinical recommendations across included guidelines.

Guideline	Timing of FP Counseling	Embryo/Oocyte Cryopreservation	GnRH-a for Ovarian Protection	Pregnancy Waiting Period	Letrozole with Ovarian Stimulation
Fertility preservation and post-treatment pregnancies in post-pubertal cancer patients: ESMO Clinical Practice Guidelines (7)	As early as possible in treatment planning; immediate referral to fertility specialist [III, A]	Established option; oocyte preferred if no partner; embryo if partner available; ≤40 years; 2-week delay feasible [III, A]	Recommended for breast cancer patients during (neo)adjuvant ChT for ovarian function preservation [I, A]; NOT alternative to cryopreservation [V, C]	At least 1 year after ChT completion [IV, B]; 3 months for tamoxifen; 7 months for trastuzumab	Recommended with letrozole to reduce estrogen levels during ovarian stimulation [III, C]
NCCN Guidelines® Insights: Breast Cancer, Version 4.2026 (9)	Refer to fertility specialist before chemotherapy and/or endocrine therapy; all premenopausal patients should be informed	Oocyte and embryo cryopreservation are explicit options	May preserve ovarian function and reduce chemotherapy-induced amenorrhea [randomized trial evidence]; conflicting results in ER-positive patients	Not mentioned	Not mentioned
Fertility preservation for medical reasons in girls and women: British fertility society policy and practice guideline (20)	As early as possible in cancer treatment pathway; shortly after diagnosis and during treatment planning [C]	Embryo: established technique [D]; Oocyte: effective technique [C]; Offered to reproductive-age women at risk of infertility	Recommended for early breast cancer [A]; other cancers may be considered [GPP]; NOT alternative to cryopreservation	Not mentioned	Recommended; consider anti-estrogen (letrozole, clomifene, or tamoxifen) during ovarian stimulation in estrogen-sensitive tumors [D]
Fertility problems: assessment and treatment (21)	Discuss before preparing for medical treatment that may impair fertility; at earliest opportunity for urgent treatment	Sperm, egg, and embryo cryopreservation are established practice; ovarian tissue cryopreservation may be considered	Not mentioned	Not mentioned	Not mentioned
No.366-Gynaecologic Management of Hereditary Breast and Ovarian Cancer (25)	BRCA carriers: urgent consultation with reproductive specialist if fertility is a concern and childbearing incomplete [strong, high]	BRCA carriers can safely undergo fertility treatments (IVF/PGD) [strong, moderate]	Not mentioned	Not mentioned	Not mentioned
Adolescent and Young Adult (AYA) Oncology, Version 2.2026 (18)	Assess and discuss before start of therapy; refer as soon as possible for interested patients	Sperm banking (male); oocyte/embryo (if 3-week delay feasible); ovarian tissue (if cannot delay treatment)	May protect ovarian function but controversial; NOT a substitute for other FP methods	Not mentioned	Not mentioned
Management of Gynecologic Issues in Women With Breast Cancer (19)	Discuss at diagnosis; refer to fertility specialist if future pregnancy desired [B]	Embryo: mature option [B]; Oocyte/ovarian tissue: investigational [C]	Evidence uncertain—may restore menses but no improvement in pregnancy rates [B]	≥12 months [B]	Letrozole + gonadotropins: no increase in recurrence at 2 years [II-2]
Clinical practice China guidelines for breast cancer during pregnancy and lactation (2025 edition) (23)	FP possible after termination of pregnancy or during cesarean section; not recommended if already received chemotherapy	Oocyte, embryo, or ovarian tissue cryopreservation after termination of pregnancy; not recommended if already received chemotherapy	Not mentioned	Not explicitly stated (focuses on timing of chemotherapy during pregnancy)	Not mentioned
Clinical practice China guidelines for fertility preservation in women of childbearing age with breast cancer (2025 edition) (24)	Communicate at diagnosis and treatment decision-making; implement before treatment initiation [I, A]	Embryo (married, I,A); Oocyte (unmarried, I,B); waiting >2 weeks before treatment; ovarian tissue (prepubertal or urgent treatment)	Recommended for premenopausal breast cancer patients during chemotherapy; NOT a substitute for cryopreservation [I, A]	MDT individualized assessment; no specific time frame specified [I, B]	Not mentioned
Fertility preservation for patients with breast cancer: The Korean Society for Fertility Preservation clinical guidelines (8)	Refer as soon as possible; early referral prevents treatment delay	Embryo: most established; Oocyte: no longer experimental; Ovarian tissue: still experimental	Controversial effectiveness; NOT a substitute for established methods	Not mentioned	Letrozole co-administration is feasible; reduces peak estradiol without decreasing oocyte yield
ESHRE guideline: female fertility preservation (10)	Provide decision aids; raise oncologists’ awareness of FP options [GPP]	Oocyte: established [strong]; Embryo: established [weak]; OTC: option when oocyte/embryo not feasible [weak]	May be offered to breast cancer patients; NOT instead of cryopreservation [weak]; not routinely recommended for other cancers	≥12 months [GPP]; uterine radiotherapy=high-risk pregnancy	Recommended in estrogen-sensitive diseases [weak]
The effects of cancer treatment on reproductive functions (22)	Fully inform prior to treatment about gonadotoxic consequences	Embryo: best validated technique; Oocyte/ovarian tissue: developing technologies, very few live births worldwide	Not mentioned	≥12 months	Not mentioned

FP, fertility preservation; ChT, chemotherapy; GnRH-a, gonadotropin-releasing hormone agonist; GPP, good practice point; MDT, multidisciplinary team; OTC, ovarian tissue cryopreservation.

### 3.5 Quality appraisal of clinical practice guideline recommendations

Beyond the content of clinical recommendations, we further evaluated the quality and implementability of these recommendations using the AGREE-REX instrument. The AGREE-REX evaluation (27) demonstrated the following mean standardized percentage scores across domains: clinical applicability achieved the highest score (73.15%), implementability received the lowest score (53.48%), while values and preferences showed intermediate performance (60.06%). Detailed evaluation results by domain are presented in Table 4.

**Table 4:** AGREE-REX evaluation results.

Guideline	Percentage of Standardization (%)			
	Clinical Applicability	Values and Preferences	Implementability	Average score
Fertility preservation and post-treatment pregnancies in post-pubertal cancer patients: ESMO Clinical Practice Guidelines (7)	88.9	100.0	100.0	96.30
Fertility preservation for patients with breast cancer: The Korean Society for Fertility Preservation clinical guidelines (8)	22.2	33.3	25.0	26.83
NCCN Guidelines® Insights: Breast Cancer, Version 4.2026 (9)	88.9	91.7	100.0	93.53
ESHRE guideline: female fertility preservation (10)	38.9	16.7	8.3	21.30
Adolescent and Young Adult (AYA) Oncology, Version 2. 2026 (18)	94.4	54.2	25.0	57.87
Management of Gynecologic Issues in Women With Breast Cancer (19)	50.0	33.3	8.3	30.53
Fertility preservation for medical reasons in girls and women: British fertility society policy and practice guideline (20)	100.0	95.8	91.7	95.83
Fertility problems: assessment and treatment (21)	100.0	95.8	91.7	95.83
The effects of cancer treatment on reproductive functions (22)	27.8	20.8	0.0	16.2
Clinical practice China guidelines for breast cancer during pregnancy and lactation (2025 edition) (23)	100.0	58.3	33.3	63.87
Clinical practice China guidelines for fertility preservation in women of childbearing age with breast cancer (2025 edition) (24)	66.7	50.0	66.7	61.13
No.366-Gynaecologic Management of Hereditary Breast and Ovarian Cancer (25)	100.0	70.8	91.7	87.50
Average score	73.15	60.06	53.47	

Clinical Applicability attained an average standardized percentage score of 73.15%. Four guidelines (20, 21, 23, 25) achieved perfect scores (100%), while two guidelines (8, 22) scored below 30%. The primary deficiencies were: (1) insufficient evaluation of evidence quality (e.g., publication bias analysis); and (2) lack of systematic elaboration on patient applicability and clinical outcome impacts.

Values and Preferences with an average standardized percentage score of 60.06%, showed two guidelines (10, 21) performing poorly (<30%). Analysis revealed: (1) inadequate consideration of local policy contexts; and (2) insufficient assessment of health equity in target populations and recommendation generalizability.

Implementability, this domain obtained the lowest average score (53.48%). Five guidelines (8, 10, 18, 19, 22) scored below 30%, primarily due to: (1) failure to evaluate required economic costs and resources for implementation; and (2) absence of specific competency training protocols. For instance, the KSFP guideline (8) recommended oocyte cryopreservation without addressing the required 2-3 week ovarian stimulation period, potential treatment delays, or financial costs, which are critical barriers in real-world clinical practice.

## 4 Discussion

This review employed a combined approach using the AGREE II and AGREE-REX instruments to conduct the first comprehensive assessment of both the methodological rigor and the implementability of recommendations within clinical practice guidelines for fertility preservation in breast cancer patients. Five guidelines (7, 9, 20, 21, 25) were rated as Grade A, primarily due to their clear definition of the scope of application, adoption of standardized evidence grading systems like GRADE, involvement of multidisciplinary teams in development, and disclosure of conflicts of interest. Most guidelines (18, 19, 23, 24) exhibited methodological limitations. Notably, two Grade C guidelines (8, 22) lacked considerations for resource requirements and economic costs, highlighting implementation challenges in clinical practice.

### 4.1 Key findings and comparisons with existing literature

This review identified significant variations in the quality of breast cancer fertility preservation guidelines across different domains in AGREE II. The “Scope and Purpose” domain demonstrated relatively higher performance (72.98%), indicating that guidelines generally emphasized defining the target health issues and populations. This finding aligns with the conclusions of Jacobs et al. (28) However, certain guidelines, such as those from ESHRE (10) and RCOG (22), exhibited issues with ambiguous descriptions of the target population. This phenomenon is particularly pronounced in breast cancer fertility preservation guidelines involving multiple disciplines, primarily due to their need to concurrently address distinct topics such as cancer treatment and reproductive health. In contrast, guidelines focused on a single specialty area, such as the ASCO guideline for male breast cancer (29), typically achieved higher scores in this domain, often attributed to their clearer definition of the target population.

Beyond methodological quality, we also compared the clinical recommendations across the included guidelines on five key clinical questions (Table 3). This comparison revealed both areas of consensus and notable discrepancies. All guidelines consistently recommended timely fertility preservation counseling and recognized embryo and oocyte cryopreservation as established options. However, substantial heterogeneity existed in several areas. The use of GnRH agonists for ovarian protection during chemotherapy was the most controversial topic: while ESMO (7), BFS (20), NCCN Guidelines (9), and the Chinese fertility preservation guideline (24) supported its use, ACOG (19) and KSFP (8) questioned its effectiveness, and other guidelines did not address it. Similarly, the recommended pregnancy waiting period after chemotherapy varied from 6 to 12 months, with the Chinese guideline (24) favoring individualized multidisciplinary assessment rather than a fixed timeframe. Recommendations regarding letrozole use during ovarian stimulation were also inconsistently reported, with several guidelines not mentioning this option. These discrepancies reflect the evolving nature of evidence in oncofertility and highlight the need for harmonized, evidence-based recommendations to reduce clinical uncertainty and improve patient counseling.

“Stakeholder Involvement” emerged as a significant weakness in the present review (54.78%), representing the lowest score among all domains. Although Armstrong et al. (30) emphasized the need to systematically include patients as stakeholders in guideline development projects to enhance clinical relevance, this review found that only three guidelines (7, 21, 23) explicitly documented patient involvement in their development processes. This substantial gap in actual participation may stem partly from the unique nature of breast cancer fertility issues, where patients facing the dual pressures of treatment and fertility-related decisions may experience reduced willingness or capacity to participate. Nevertheless, a more critical factor appears to be the persistence of an “expert-driven” mindset within some guideline development groups, potentially leading to insufficient recognition of the unique value patients bring in defining health issues, clarifying values and preferences, and identifying implementation barriers. Notably, the NICE guideline (21), which demonstrated high patient involvement, explicitly documenting patient involvement through public consultations and focus groups, achieved a score of 95.8% in the “Values and Preferences” domain, whereas the ESHRE guideline (10), lacking documented patient participation, scored only 16.7%. This stark contrast further underscores the critical role of patient involvement in enhancing guideline quality (31). This was further illustrated by our sensitivity analysis, in which the NCCN Guidelines (9) was downgraded from Grade A to B under stricter thresholds due to a marginal Stakeholder Involvement score (65.3%), despite excellent performance in other domains. This observation underscores both the importance of stakeholder engagement and the inherent sensitivity of threshold-based classifications.

The “Rigor of Development” domain yielded a relatively low average score (55.30%), indicating prevalent methodological limitations within the assessed guidelines. A primary concern was the inadequacy of updating mechanisms. Only four guidelines (7, 9, 21, 25) explicitly committed to a defined update cycle, falling short of international standards stipulating that guidelines should typically be updated every three years to ensure the currency of evidence (32). This absence of a formal update process risks rendering guideline recommendations obsolete relative to evolving clinical practice. For instance, the 2007 RCOG guideline (22), which had not undergone revision for an extended period, still recommended a 12-month post-treatment contraception period; in contrast, the 2026 NCCN Guidelines (9) has reduced this recommended duration to 6 months. Secondly, insufficient transparency regarding evidence grading and recommendation strength significantly undermined the scientific credibility of several guidelines. For example, the KSFP guideline (8) failed to describe its methodology for grading evidence, resulting in recommendations lacking traceable scientific justification and an inability to differentiate between conclusions based on expert consensus vs. those supported by higher levels of evidence.

The “Applicability” domain achieved an average score of 69.33%. Eight guidelines (7, 9, 10, 19, 21–23, 25) scored above 60%, with a key strength being the integration of implementation frameworks for multidisciplinary team collaboration. However, a significant weakness observed in the vast majority of guidelines was inadequate consideration of resource implications. This was most notably reflected in the lack of assessment regarding the availability of critical resources (such as fertility preservation technologies), cost-effectiveness analyses, and pathways for insurance coverage. Given that breast cancer fertility management is heavily reliant on such high-cost, technologically intensive resources, this widespread neglect of resource allocation considerations aligns closely with the findings of Zhou et al. (33), who identified resource constraints as the primary implementation barrier. This underscores that even with robust collaborative frameworks in place, the absence of realistic resource assessment and corresponding mitigation strategies will pose significant obstacles to the successful implementation of guidelines in clinical practice.

The “Editorial Independence” domain achieved an average score of 71.81%, yet exhibited significant polarization among the guidelines. Six guidelines (7–9, 20, 21, 25) attained perfect scores in disclosing conflicts of interest, demonstrating a high degree of transparency. Conversely, the ACOG (19) and RCOG (22) guidelines provided no information regarding conflicts of interest whatsoever, resulting in scores of zero for this domain. This discrepancy suggests a tendency towards selective transparency in some guideline development processes, potentially avoiding the disclosure of information related to sensitive commercial interests or significant personal conflicts (34). A systematic analysis of funding sources and their potential influence on recommendation bias was beyond the scope of this review; however, no overt discrepancies were observed between funding declarations and the directionality of the recommendations. Particularly within the commercially sensitive field of fertility management, complete and transparent disclosure of conflicts of interest remains an indispensable cornerstone for safeguarding the scientific credibility of guidelines (35).

It is worth noting that the relatively lower scores of the two Chinese guidelines (23, 24) primarily reflect deficiencies in methodological reporting, such as limited documentation of stakeholder involvement and editorial independence, which may be partly attributable to different reporting standards and resource contexts, rather than necessarily indicating inferior clinical content.

### 4.2 Implications for practice and policy

Based on the findings presented above, this review proposes the following key recommendations for the future development, dissemination, and implementation of breast cancer fertility preservation guidelines: First, stakeholder involvement, particularly patient engagement, must be strengthened. Guideline development groups must integrate patients, especially breast cancer survivors who have navigated fertility-related decisions as core members. These patient representatives should be granted voting rights during critical phases, including topic prioritization and recommendation formulation. Furthermore, structured questionnaires should be systematically employed to collect and quantify patient needs, values, and preferences. This entire process must be explicitly documented within the guideline itself. Such measures will significantly enhance the clinical relevance and patient acceptability of the guidelines. Second, robust methodological processes and dynamic updating mechanisms must be established and maintained. Development groups should strictly adhere to established guideline development standards, detailing the processes for evidence retrieval, appraisal, and recommendation formulation. A publicly committed update cycle should be established. Furthermore, the GRADE framework should be employed dynamically to annotate the impact of new evidence on the current grades of recommendations in a timely manner. This ensures that guideline recommendations remain synchronized with the latest clinical evidence and avoids obsolescence.

Third, implementation strategies and resource toolkits must be integrated to provide actionable support. This includes embedding resource assessment checklists within guideline appendices, developing clear flowcharts for multidisciplinary team collaboration, and providing strategies to address potential barriers. This comprehensive approach is critical for facilitating guideline implementation, particularly in resource-constrained settings. Finally, transparency and editorial independence must be ensured. All guidelines must clearly and comprehensively disclose funding sources and conflicts of interest for all participants. Additionally, a public declaration affirming that the editorial process was free from funder influence is essential. Adherence to international standards established by organizations such as the Guidelines International Network (GIN) (36) and the Institute of Medicine (IOM) (37) is paramount for maintaining the credibility of the guidelines. Furthermore, cultural adaptation is particularly critical when implementing Western-developed guidelines in Asian contexts. For example, while Western guidelines generally recommend immediate fertility counseling upon diagnosis, discussing reproductive issues concurrently with a new cancer diagnosis may be perceived as taboo or may cause additional psychological distress in some Asian cultural settings. A phased, culturally sensitive approach—such as providing initial emotional support followed by deferred fertility discussions once the patient has processed the diagnosis—may be more acceptable and effective in these populations.

### 4.3 Limitations and future directions

While this review provides valuable insights into the quality of clinical practice guidelines for fertility preservation in breast cancer patients, several limitations should be acknowledged. First, the review focused solely on guidelines published in Chinese and English, potentially excluding high-quality guidelines in other languages. Future research should consider multilingual evaluations to provide a more comprehensive analysis. Second, the AGREE II instrument does not prescribe specific cut-off scores for guideline quality, leaving grading decisions to the discretion of researchers. Although the thresholds of 60% and 30% were adopted based on common practices observed in prior guideline appraisal studies, the grading system used in this review (Grades A, B, and C) remains researcher-defined and inherently subjective; different thresholds might yield different classifications, and our results should be interpreted with this consideration in mind (17). Our sensitivity analysis confirmed this concern: when stricter thresholds (70% for Grade A and 40% for Grade C) were applied, the NCCN Guidelines (9) was downgraded from A to B due to a marginal Stakeholder Involvement score (65.3%), illustrating that guidelines near threshold boundaries may be susceptible to the choice of cut-off values. Furthermore, the relatively lower AGREE II scores of the two Chinese guidelines (23, 24) should be interpreted with caution. AGREE II primarily evaluates the methodological quality and transparency of the guideline development process; it does not directly determine whether individual clinical recommendations are correct, evidence-based, or clinically valid. Therefore, lower AGREE II scores should not be equated with lower clinical validity or inferior recommendations. This distinction is particularly important when comparing guidelines developed in different healthcare systems and sociocultural contexts, where reporting standards and development resources may vary considerably. Finally, the review did not evaluate the clinical effectiveness of the recommendations, which is a critical aspect of guideline utility. Future studies should integrate guideline quality assessments with evaluations of their impact on patient outcomes.

## 5 Conclusion

This study presents the first comprehensive evaluation of twelve breast cancer fertility preservation guidelines using combined AGREE II and AGREE-REX instruments, revealing significant quality variations across key domains, particularly Stakeholder Involvement, Rigor of Development, and Applicability. Sensitivity analysis confirmed the robustness of these findings, although classifications of borderline guidelines should be interpreted with caution. Addressing the identified methodological flaws can enhance recommendation reliability and applicability, empowering clinicians and patients to make evidence-based decisions. Although five high-quality guidelines were identified as clinical core references, all are available only in English. Thus, prior to implementation, these guidelines require translation, cultural adaptation to align with the specific contexts and healthcare systems of target countries, and clinical validation.

## Data Availability

The original contributions presented in the study are included in the article/Supplementary Material, further inquiries can be directed to the corresponding author.
