# Supplementary material for: Quality appraisal of clinical practice guidelines for fertility preservation in patients with breast cancer: a systematic review using AGREE II and AGREE-REX
Source: Front Med (Lausanne). 2026 Oct 7;13:1930322. doi: 10.3389/fmed.2026.1930322 (PMC13683367; doi:10.3389/fmed.2026.1930322)
Supplement: Supplementary file 2 [file Table2.docx]

**Supplementary Material 2**——Search formula and related information

| **Pubmed** | | |
| --- | --- | --- |
| #1 | (((((("Breast Neoplasm*"[MeSH Terms])OR("Breast tumor"[Title/Abstract]))OR("Breast carcinoma"[Title/Abstract]))OR("Mammary cancer"[Title/Abstract]))OR("Mammary carcinoma"[Title/Abstract]))OR("Mammary neoplasm"[Title/Abstract]))OR("Mammary tumor"[Title/Abstract]) | 3,92308 |
| #2 | (((((("Fertility*"[MeSH Terms])OR("Fecundability"[Title/Abstract]))OR("Fecundity"[Title/Abstract]))OR("Fertility Incentive*"[Title/Abstract]))OR("Marital Fertility"[Title/Abstract]))OR("Natural Fertility"[Title/Abstract]))OR("World Fertility Survey"[Title/Abstract]) | 67865 |
| #3 | (((guidelines[Title])OR(clinical pratical guidelines[Title]))OR(CPGs[Title]))OR(Practice Guideline[Title]) | 87373 |
| #4 | #1 AND#2 AND#3 | 107 |
| **Web of science** | | |
| #1 | TI=(Breast Neoplasm*OR Breast tumor OR Breast carcinoma OR Mammary cancer OR Mammary carcinoma OR Mammary neoplasm OR Mammary tumor) | 133,217 |
| #2 | TI=(Fertility*OR Fecundability OR Fecundity OR Fertility Incentive*OR Fertility Incentive*OR Natural Fertility OR World Fertility Survey) | 100,284 |
| #3 | guidelines(Topic)or clinical pratical guideline(Topic)or CPGs(Topic)or Practice Guideline(Topic) | 903,105 |
| #4 | #1 AND#2 AND#3 | 96 |
| **Cochrane Library** | | |
| #1 | (Breast Neoplasm*OR Breast tumor OR Breast carcinoma OR Mammary cancer OR Mammary carcinoma OR Mammary neoplasm OR Mammary tumor):ti,ab,kw | 33,276 |
| #2 | (Fertility*OR Fecundability OR Fecundity OR Fertility Incentive*OR Marital Fertility OR Natural Fertility OR World Fertility Survey):ti,ab,kw | 5,872 |
| #3 | (guidelines*OR clinical pratical guideline OR CPGs OR Practice Guideline):ti,ab,kw | 61,762 |
| #4 | #1 AND#2 AND#3 | 287 |
| **Embase** | | |
| #1 | (Breast Neoplasm* or Breast Tumor* or Breast Carcinoma or Breast Cancer or Mammary Neoplasm* or Mammary Tumor* or Mammary Carcinoma or Mammary Cancer).mp. [mp=title, abstract, heading word, drug trade name, original title, device manufacturer, drug manufacturer, device trade name, keyword heading word, floating subheading word, candidate term word] | 847353 |
| #2 | (Fertility* or Fecundability or Fecundity or "Fertility Incentive*" or "Marital Fertility" or "Natural Fertility" or "World Fertility Survey").mp. [mp=title, abstract, heading word, drug trade name, original title, device manufacturer, drug manufacturer, device trade name, keyword heading word, floating subheading word, candidate term word] | 200838 |
| #3 | ("Clinical Practice Guideline" or "Clinical Practice Guidelines" or CPGs or ("Practice Guideline" or "Practice Guidelines")).mp. [mp=title, abstract, heading word, drug trade name, original title, device manufacturer, drug manufacturer, device trade name, keyword heading word, floating subheading word, candidate term word] | 657086 |
| #4 | #1 AND #2 AND #3 | 246 |
| **Scopuse** | | |
| #1 | (TITLE-ABS-KEY("Breast Neoplasm" OR "Breast Tumor" OR "Breast Carcinoma" OR "Breast Cancer") OR  TITLE-ABS-KEY("Mammary Neoplasm" OR "Mammary Tumor" OR "Mammary Carcinoma" OR "Mammary Cancer")) | 739927 |
| #2 | (TITLE-ABS-KEY(Fertility)OR TITLE-ABS-KEY(Fecundability)OR TITLE-ABS-KEY(Fecundity)OR TITLE-ABS-KEY(Fertility AND Incentive)OR OR TITLE-ABS-KEY(Natural AND Fertility)) | 294843 |
| #3 | (TITLE-ABS-KEY(clinical AND practical AND guidelines)OR TITLE-ABS-KEY(guidelines)OR TITLE-ABS-KEY(CPGs)OR TITLE-ABS-KEY(Practice Guideline)) | 118204 |
| #4 | #1 AND #2 AND #3 | 537 |
| **CNKI** | | |
| #1 | SU= '乳腺癌' OR SU %= '乳腺肿瘤' OR SU %= '乳癌' OR SU %= '乳腺恶性肿瘤' | 12640000 |
| #2 | SU= '生育管理' OR SU %= '生育服务' OR SU %= '生育健康' OR SU %= '生育行为' | 1200000 |
| #3 | SU= '临床实践指南' OR SU %= '指南' | 7750000 |
| #4 | #1 AND#2 AND#3 | 1129 |
| **WangFang** | | |
| #1 | （题名或关键词:(乳腺癌)or题名或关键词:(乳腺肿瘤)or题名或关键词:(乳癌)or题名或关键词:(乳腺恶性肿瘤）） | 13510000 |
| #2 | （题名或关键词:(生育管理)or题名或关键词:(生育服务)or题名或关键词:(生育健康)or题名或关键词:(生育行为）or题名或关键词:(生育意愿）） | 1165400 |
| #3 | （题名或关键词:(临床实践指南)or题名或关键词:(指南)) | 7,948,736 |
| #4 | #1 AND#2 AND#3 | 675 |
| **VIP** | | |
| #1 | M=乳腺癌OR乳腺肿瘤OR乳癌OR乳腺恶性肿瘤 | 183210000 |
| #2 | M=生育管理OR生育服务OR生育健康OR生育行为OR生育意愿 | 37650000 |
| #3 | M=临床实践指南OR指南 | 219,570,000 |
| #4 | #1 AND#2 AND#3 | 452 |
