# Supplementary material for: Quality appraisal of clinical practice guidelines for fertility preservation in patients with breast cancer: a systematic review using AGREE II and AGREE-REX
Source: Front Med (Lausanne). 2026 Oct 7;13:1930322. doi: 10.3389/fmed.2026.1930322 (PMC13683367; doi:10.3389/fmed.2026.1930322)
Supplement: Supplementary file 3 [file Table4.docx]

**Supplementary Material 4**——Excluded studies and reasons

***-Population not targeted patients with breast cancer.***

1. Borderline Ovarian Tumors: French Guidelines from the CNGOF. Part 1. Epidemiology, Biopathology, Imaging and Biomarkers
2. Evidence based management of patients with endometriosis undergoing assisted conception: British fertility society policy and practice recommendations
3. Multidisciplinary consensus on the criteria for fertility preservation in cancer patients
4. Fertility preservation for patients with hematologic malignancies: The Korean Society for Fertility Preservation clinical guidelines
5. Fertility preservation in patients undergoing gonadotoxic treatments: a Canadian Fertility and Andrology Society clinical practice guideline
6. Guideline No. 446: Hysteroscopic Surgery in Fertility Therapy
7. Severe maternal morbidity: screening and review.
8. Guideline No. 435: Minimally Invasive Surgery in Fertility Therapy
9. 多囊卵巢综合征相关不孕治疗及生育保护共识

***-Old version of guideline***

1. Fertility preservation and post-treatment pregnancies in post-pubertal cancer patients: ESMO Clinical Practice Guidelines
2. Adolescent and Young Adult (AYA) Oncology, Version 2.2024, NCCN Clinical Practice Guidelines in Oncology
3. NCCN Guidelines Version 4.2024 Breast Cancer
4. NCCN Guidelines® Insights: Breast Cancer, Version 4.2023
5. NCCN Guidelines Version 4.2022 Breast Cancer
6. NCCN Guidelines® Insights: Breast Cancer, Version 4.2021
7. NCCN Guidelines® Insights: Breast Cancer, Version 4.2020
8. Adolescent and young adult oncology. Clinical practice guidelines in oncology

***-Comments or letter***

1. *New Recommendations for Breast Cancer Screening-In Pursuit of Health Equity Guide interpretation*
2. *Breast cancer guidelines in action.*
3. *Clinical Breast Examination and Breast Cancer Screening Guideline--Reply*
4. *Is guideline adherence the ke*y to increased survival in patients with breast cancer?

***-Guide interpretation***

1. 女性生育力保存ESHRE 2020年指南解读
2. 2023 ESGO/ESHRE/ESGE子宫内膜癌保留生育功能治疗指南解读
3. NCCN临床实践指南：乳腺癌（2025.V3）—更新解读

***-Guideline translation***

1. NCCN乳腺癌临床实践指南中文版更新
2. NCCN临床实践指南：降低乳腺癌风险（2021.V1）-中文版
3. 乳腺癌预防性治疗专家共识（摘译版）
