# Supplementary material for: Quality appraisal of clinical practice guidelines for fertility preservation in patients with breast cancer: a systematic review using AGREE II and AGREE-REX
Source: Front Med (Lausanne). 2026 Oct 7;13:1930322. doi: 10.3389/fmed.2026.1930322 (PMC13683367; doi:10.3389/fmed.2026.1930322)
Supplement: Supplementary file 4 [file Table3.doc]

**Supplementary Material 3** Sensitivity Analysis of Guideline Classifications under Alternative AGREE II Threshold Criteria

**1 Background**

The AGREE II instrument does not provide official cut-off values for distinguishing guideline quality levels, requiring researchers to predefine thresholds based on the study context. To assess whether the choice of threshold values influenced our guideline classifications, we conducted a sensitivity analysis using an alternative grading criteria with stricter thresholds (70% for Grade A and 40% for Grade C).

**2 Methods**

**2.1 Original Classification Criteria**

- Grade A (Strongly Recommended): Guidelines that achieved standardized percentage scores above 60% in all six domains.
- Grade B (Recommended with Revisions): Guidelines that scored between 30% and 60% in more than three domains.
- Grade C (Not Recommended): Guidelines that received standardized percentage scores below 30% in more than three domains.

**2.2 Sensitivity Analysis Criteria**

- Grade A (Strongly Recommended): Guidelines that achieved standardized percentage scores above 70% in all six domains.
- Grade B (Recommended with Modifications): Guidelines that scored between 40% and 70% in more than three domains.
- Grade C (Not Recommended): Guidelines that received standardized percentage scores below 40% in more than three domains.

**3 Results**

Table S1 presents the comparison of guideline classifications between the original criteria and the sensitivity analysis.

Table S1 Comparison of guideline classifications between original and alternative AGREE II threshold criteria

| **Guideline** | **Original Classification** | **Sensitivity Analysis** |
| --- | --- | --- |
| Fertility preservation and post-treatment pregnancies in post-pubertal cancer patients: ESMO Clinical Practice Guidelines^Error: Reference source not found^ | A | A |
| NCCN Guidelines® Insights: Breast Cancer, Version 4.2026^Error: Reference source not found^ | A | B |
| Fertility preservation for medical reasons in girls and women: British fertility society policy and practice guideline^Error: Reference source not found^ | A | A |
| Fertility problems: assessment and treatment^Error: Reference source not found^ | A | A |
| No.366-Gynaecologic Management of Hereditary Breast and Ovarian Cancer^Error: Reference source not found^ | A | A |
| Adolescent and Young Adult (AYA) Oncology, Version 2.2026^Error: Reference source not found^ | B | B |
| Management of Gynecologic Issues in Women With Breast Cancer^Error: Reference source not found^ | B | C |
| Clinical practice China guidelines for breast cancer during pregnancy and lactation (2025 edition)^Error: Reference source not found^ | B | B |
| Clinical practice China guidelines for fertility preservation in women of childbearing age with breast cancer (2025 edition)^Error: Reference source not found^ | B | B |
| Fertility preservation for patients with breast cancer: The Korean Society for Fertility Preservation clinical guidelines^Error: Reference source not found^ | C | C |
| ESHRE guideline: female fertility preservation^Error: Reference source not found^ | C | C |
| The effects of cancer treatment on reproductive functions^Error: Reference source not found^ | C | C |

**4 Summary of Sensitivity Analysis Findings**

Under the stricter threshold criteria (70% for Grade A, 40% for Grade C):

4.1 10 of 12 guidelines (83.3%) retained their original classifications.

4.2 Two guidelines were downgraded:

- NCCN Breast [9]: A → B. This guideline scored 65.3% in the Stakeholder Involvement domain, slightly below the 70% threshold for Grade A. Notably, it scored above 71% in all other five domains (range: 71.4%–100%) and was rated as High quality by AGREE-REX. This downgrade primarily reflects the sensitivity of threshold selection for borderline cases rather than a fundamental quality deficit of the guideline.
- ACOG [19]: B → C. This downgrade was attributable to scores below 40% in two domains: Editorial Independence (0%) and Rigor of Development (33.3%), reflecting well-documented methodological deficiencies.

**5 Conclusion**

The sensitivity analysis confirms the overall robustness of our core findings. Among the five guidelines initially rated as Grade A, all retained this classification except NCCN Breast [9], which was downgraded to Grade B due to a marginal shortfall in Stakeholder Involvement (65.3% vs. the 70% threshold). Critically, no C-grade guideline was upgraded to A, and no A-grade guideline was downgraded to C, underscoring the reliability of our system in discriminating between high- and low-quality guidelines. The observed downgrades were attributable to either borderline threshold effects (NCCN Breast [9]) or clearly identifiable methodological deficiencies (ACOG [19]), rather than systemic instability, further supporting the consistency of our grading approach.
